# *OsSPL88* Encodes a Cullin Protein that Regulates Rice Growth and Development

**DOI:** 10.3389/fgene.2022.918973

**Published:** 2022-07-11

**Authors:** Zhengai Chen, Wenjing Yin, Xuan Li, Tao Lu, Hanfei Ye, Gaoxing Dai, Yijian Mao, Sanfeng Li, Penggen Duan, Mei Lu, Yuchun Rao, Yuexing Wang

**Affiliations:** ^1^ College of Chemistry and Life Sciences, Zhejiang Normal University, Jinhua, China; ^2^ Guangxi Academy of Agricultural Sciences, Nanning, China; ^3^ State Key Laboratory of Rice Biology, China National Rice Research Institute, Hangzhou, China

**Keywords:** lesion mimics, cullin protein, SPL88, growth and development, rice

## Abstract

Plant lesion mimics refer to necrotic spots spontaneously produced by the plant without mechanical damage, pathogen invasion, and adversity stress. Here, we isolated and characterized two rice (*Oryza sativa* L) mutants, namely, *spl88-1* (*spotted leaf88*-*1*) and *spl88-2* (*spotted leaf88*-*2*), which were identified from an ethyl methanesulfonate-mutagenized *japonica* cultivar Xiushui 11 population. Physiological and biochemical experiments indicated that more ROS accumulated in *spl88-1* and *spl88-2* than in wild type. *spl88-1* and *spl88-2* displayed spontaneous cell death and enhanced their resistance to bacterial blight by affecting the expression of defense-related genes. We isolated *SPL88* by map-based cloning, which encoded a highly conserved Cullin protein. A single base deletion was detected in *spl88-1* and *spl88-2*, in which the 132nd base C of *SPL88-1* and the 381th base T of *SPL88-2* were deleted, causing premature termination of protein translation. *SPL88* was expressed in root, stem, leaf, leaf sheath, and panicle. The Cullin protein was localized in the cytoplasm and nucleus. The aforementioned results indicate that *SPL88* regulates the growth and development of rice by affecting the expression of defense-related genes.

## Introduction

Plant lesion mimics are the spontaneous formation of necrotic spots on leaves and sheath (even stem and seed) in abiotic or biotic stress (Cai et al., 2021). This process involves programmed cell death (PCD) (Huang et al., 2010; McGrann et al., 2015), and the spot area can reflect the degree of cell death (Shirasu and Pual, 2000). According to the developmental process of the lesion mimics, it can be divided into the whole life lesion mimics, such as *lrd32*, *lrd35*, and *lrd40*.; vegetative initiation lesion mimics, such as *lrd31*, *lrd41*, and *lrd44*; and reproductive initiation lesion mimics, such as *lrd27, lrd28* and *lrd39* (Wang et al., 2004).

The mechanism underlying the generation of lesion mimics is very complex, mainly due to resistance-related gene mutations, chlorophyll metabolism disorders, programmed cell death defects, and changes in signal pathways (Takahashi et al., 1999; Wu et al., 2008). The mutation of the *NLS1* gene encoding CC-NB-LRR protein blocks the independent defense signaling pathway between salicylic acid (SA) and *NPR1*, causing a large accumulation of SA and hydrogen peroxide (H_2_O_2_), resulting in lesion mimics on rice leaf sheaths (Tang et al., 2011). *Les22* encodes a key enzyme in the chlorophyll biosynthesis pathway, uroporphyrinogen decarboxylase (UROD). After mutation, it leads to the accumulation of its substrate uroporphyrin and induces lesion mimics on maize leaves (Hu et al., 1998). *LSD1P* encodes a zinc finger protein that negatively regulates programmed cell death depending on the accumulation of SA and the expression of *NIM1*/*NPR1* (Aviv et al., 2002). Copines are a calcium-dependent phospholipid binding protein *Arabidopsis* mutant *cpn1-1* that inhibits the production of lesion mimics in high humidity, indicating that the primary function of *CPN1* may be the regulation of plant responses to low humidity (Jambunathan et al., 2001).

The current study shows that since not all lesion mimic mutants are caused by mutations in disease resistance–related genes, most of them have significantly increased disease resistance (Mizobuchi et al., 2002). In 1965, the lesion mimic mutant *spl1* was first discovered in the rice cultivar Asahi (Liu et al., 2004), which enhanced blast resistance and was accompanied with a high expression of defense-related genes. Wu et al. (Wu et al., 2008) obtained 21 lesion mimic mutants through four different mutagenesis methods and found *spl21* and *spl24* had bacterial blight enhanced resistance and provided essential materials for dissection of the disease resistance pathways in rice. To further explore the relationship between PCD and disease resistance, Yin et al. (Yin et al., 2000) found that *spl11* leaf lesion mimics were caused by apoptosis through trypan blue staining, and *spl11* had non-race-specific resistance. It means not only resistance to bacterial blight but also against blast, providing clues for developing broad-spectrum resistance to multiple pathogens.

The Cullin protein family has a conserved CULLIN homology domain, which can bind itself as a molecular scaffold to the RING E3 ubiquitin ligase to form the Cullin-RING E3 ubiquitin ligase complex (CRL); the ubiquitin/proteasome pathway plays an important role in growth and development (Zeng et al., 2004; Figueroa et al., 2005) and defense responses (Gao et al., 2020). In the mouse model, Cullin3 plays an indispensable role in early embryogenesis and cell division cycle (Singer et al., 1999). In *Arabidopsis* thaliana, Cullin3a and Cullin3b are functionally redundant, but the absence of Cullin3a and Cullin3b affects endosperm development and leads to embryo death (Thomann et al., 2005). Jin et al. (Zhao et al., 2014) found that DWARF3, a nuclear-localized protein of F-box, was rich in leucine repeats, interacts with *OsCcullin1* to form an SCF complex (comprising Skip, Cullin, and F-box), and binds DWARF14 to inhibit branching of rice shoots. *OsCUL3a* interacts with *OsRBX1a* and *OsRBX1b* in rice to form a CUL loop-like E3 ubiquitin ligase complex, which depends on the 26s proteasome to degrade *OsNPR1* and negatively regulate plant immunity and cell death (Liu et al., 2017).

Here, we described two rice lesion mimic mutants that exhibited programmed cell death and increased resistance to bacterial blight. On the basis of map-based cloning and complementation research studies, we demonstrated that the phenotype of the mutants was caused by a mutation in *SPL88*.

## Materials and Methods

### Plant Material and Population Construction

The spotted leaf mutants *spl88-1* and *spl88-2* were isolated from a methanesulfonate (EMS)-induced mutant library of Xiushui 11 rice (wild type, WT). Two mutants were hybridized with NJ06 as the male parent, and the F_1_ offspring and the F_2_ population were grown in the rice experimental field of Zhejiang Normal University, Jinhua City, Zhejiang Province, China, during the summer of 2020 and 2021. The F_2_ populations of both *spl88-1*/NJ06 and *spl88-2*/NJ06 were used for genetic analysis, and the F_2_ recessive individuals of *spl88-1*/NJ06 and *spl88-2*/NJ06 were used for gene mapping. The agronomic traits of WT, *spl88-1*, and *spl88-2* were statistically analyzed, including plant height, tiller number, number of branches, grain number per panicle, 1000-grain weight, and seed setting rate. These results were analyzed based on the average of 10 replicates. The relevant data were statistically analyzed by SPSS 20.0 software, and graphs were plotted by GraphPad Prism 7 software.

### Shading Assay

At the tillering stage, when the mutants just appeared the lesion mimicked the phenotype. WT, *spl88-1*, and *spl88-2* were subjected to shading experiments and used about 2 cm of tin foil to shade the leaves where no lesion mimics appeared. Observations, photographs, and comparisons were made after 7 days.

### Measuring Photosynthetic Parameters and Chlorophyll Content

At noon in summer, ten WT, *spl88-1*, and *spl88-2* with relatively uniform growth vigor were selected to measure with an LI-6400XT portable photosynthesis tester at the tillering stage. Three biological replicates were performed for each measurement.

Flag leaves of wild type, *spl88-1*, and *spl88-2* were sampled separately for chlorophyll determination. The leaves were weighed, and 0.2 g of leaf tissue was cut into pieces, soaked in 95% alcohol, wrapped in tin foil and stored at 4°C for 24 h, and inverted up and down every 8 h until the green of the leaves was completely faded. The absorbance values at 470 nm, 645, and 663 nm were measured by a spectrophotometer, and the photosynthetic pigment content was calculated by the method of Lichtenthaler (Lichtenthaler, 1987). Three biological replicates were performed for each group and statistically analyzed by Student’s *t*-test.

Chlorophyll a(Chl a)concentration = 13.95A665-6.88A649.

Chlorophyll b(Chl b)concentration = 24.96A649-7.32A665.

Carotenoid(Car)concentration =(1000A470−2.05Ca−114Cb)/245.

### Electron Microscope Observation and Histochemical Analysis

At the tillering stage, WT and mutant plants with relatively uniform growth vigor were selected for scanning electron microscope (SEM) and transmission electron microscope (TEM) observation. The experimental method was referred to Rao (Rao, 2016). The content of hydrogen peroxide (H_2_O_2_), reactive oxygen species (ROS) and malondialdehyde (MDA) and the enzymatic activity of superoxide dismutase (SOD) and peroxidase (POD) were analyzed under the instructions of the manufacturer. (Nanjing Jiancheng Bioengineering Institute, Nanjing, China). TdT-mediated dUTP Nick-End Labeling (TUNEL) was used to detect DNA fragmentation and determine the degree of apoptosis. At the tillering stage, the leaves of the WT, *spl88-1*, and *spl88-2* were taken and placed in a centrifuge tube filled with 2 ml of FAA fixative (Liang and Zhou, 2018) and the centrifuge tube was pumped into a vacuum until the tissue sample was completely sunk to the bottom of the tube. An apoptosis detection kit was used to measure apoptosis in the samples (Inada et al., 1998).

### Inoculation Test

*Xanthomonas oryzae pv. Oryzae, Xoo* (the causal agent of bacterial blight) was inoculated onto the fresh leaf, free of disease spots, and senescence flag leaves of WT, *spl88-1*, and *spl88-2* at the tillering stage. Specifically, the tip of each green and healthy rice leaf (∼1.5 cm) was cut with scissors that was dipped into bacterial blight solution before making each cut. The phenotype was observed 15 days after inoculation. Moreover, the length of the lesion was photographed and measured.

### Quantitative Reverse-Transcription PCR Analysis

Root, stem, leaf, leaf sheath, and panicle samples were collected from WT and mutant plants at each stage of development. Total RNA of rice was extracted from the samples by an RNAprep pure Plant Kit (Cat No. DP432, Tiangen Biotech, Beijing, China), amplified using a ReverTra Ace^®^ qPCR RT Master with a+ gDNA Remover kit (Code: FSQ-301, TOYOBO, Japan) and backup for post-reverse transcription. Reverse-transcription PCR (qRT-PCR) was used to detect the expression of defense-related genes in mutant plants and the expression of *SPL88* in tissues, with the *OsActin* gene used as an internal reference (GenBank accession number: NM001058705). The reaction mixtures contained 1 μl cDNA template, 5 μl 2 × SYBR qPCR mix, 1 μl each of forward and reverse primers, and ddH_2_O to a final volume of 10 μl. The reaction program was 98°C for 30 s; 98°C for 5 s, 55°C for 10 s; and 72°C for 5 s for 40 cycles (Jozefczuk and Adjaye, 2011). Each reaction was performed in triplicate, using the 2^−ΔΔCt^ method for calculations. RT-PCR was performed using a quantitative fluorescence gene amplification instrument (qTOWER3G; Jena, Germany). Data were analyzed by Excel and SPSS 20.0 software. Student’s *t*-test was used to analyze the significance of the differences. The primers used for qRT-PCR are shown in Supplementary Table S1.

### Vector Construction

The complete genomic DNA fragment (including the promoter) of WT *SPL88* was amplified by PCR with primers *SPL88-1300-F/SPL88-1300-R* and then ligated with the digested binary vector pCAMBIA1300 by the Clontech In-Fusion PCR (TaKaRa) kit to construct complementary vectors of mutants *spl88-1* and *spl88-2*. The primer pair *SPL881305.1-F/SPL88-1305.1-R* was used to amplify the promoter sequence of the *SPL88* gene, and the sequence was ligated with the binary vector pCAMBIA1305.1 to obtain the GUS vectors, investigating the tissue expression of *SPL88* genes. The full-length *SPL88* open-reading frame (ORF) was amplified with the primer pair *SPL88-1132-F/SPL88-1132-R*, and the coding sequence of *SPL88* was inserted into the binary vector pYBA1132 containing the 35S promoter (*p35S::SPL88*) for subcellular localization analysis. The primers used for vector construction are shown in Supplementary Table S2.

### Transcriptome Sequencing and data Analysis

At the tillering stage, flag leaves of wild-type, *spl88-1*, and *spl88-2* were sampled, and RNA extraction was performed with an RNAprep pure Plant Kit (Cat No. DP432, Tiangen Biotech, Beijing, China). Illumina HiSeq (Illumina, San Diego, CA, United States) library construction was performed according to the manufacturer’s instructions. Differentially expressed genes (DEGs) were detected by DESeq (http://www-huber.embl.de/users/anders/DESeq/) programs. Gene function was analyzed with the KEGG analysis tool (http://www.genome.jp/kegg/). Sequencing and bioinformatics services were completed by Shanghai Paisenuo Biotechnology Co., Ltd (China).

## Result

### Phenotype of the Lesion Mimic Mutants *spl88-1* and *spl88-2*

**FIGURE 1:**
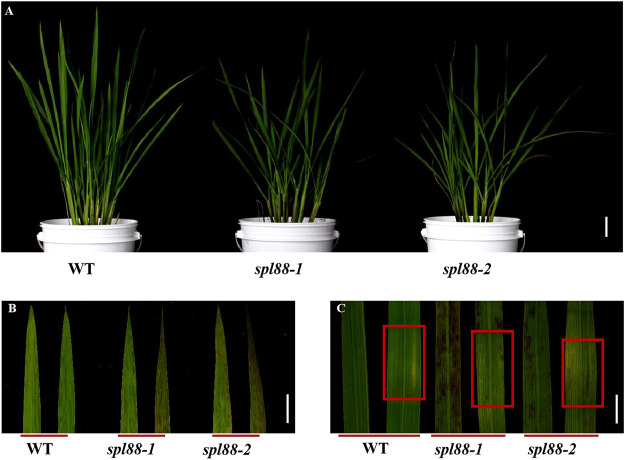
Phenotypic analysis of the wild type and mutant plants. **(A)** Phenotype diagram of wild type and mutant plants; Bar = 15 cm. **(B)** Leaf phenotype of wild type and mutant plants; Bar = 3 cm. **(C)** Phenotype of wild type and mutant plants before and after shading; Bar = 2 cm.

Under normal planting conditions in the summer field, the leaves of *spl88-1* and *spl88-2* were not significantly different from those of WT (Figure 1A) before the tillering stage. During the tillering stage, the necrotic spots appeared from the apex of the leaf, and from the tillering stage to the heading stage, the necrotic spots became more serious and spread to the whole leaf (Figure 1B). The phenotype of most rice lesion formation is induced by light (Livak and Schmittgen, 2001). During the tillering stage, *spl88-1* and *spl88-2* were subjected to shading experiments, and some newly emerged leaves were covered with 2–3 cm of aluminum foil and their phenotype was observed after 7 days. It was found that no lesions were produced in the area covered by tinfoil, but the lesions spread on the uncovered leaves (Figure 1C). These results indicate that *spl88-1* and *spl88-2* developed lesions in a light-dependent manner.

### Agronomic Traits of the Lesion Mimic Mutants *spl88-1* and *spl88-2*

**FIGURE 2:**
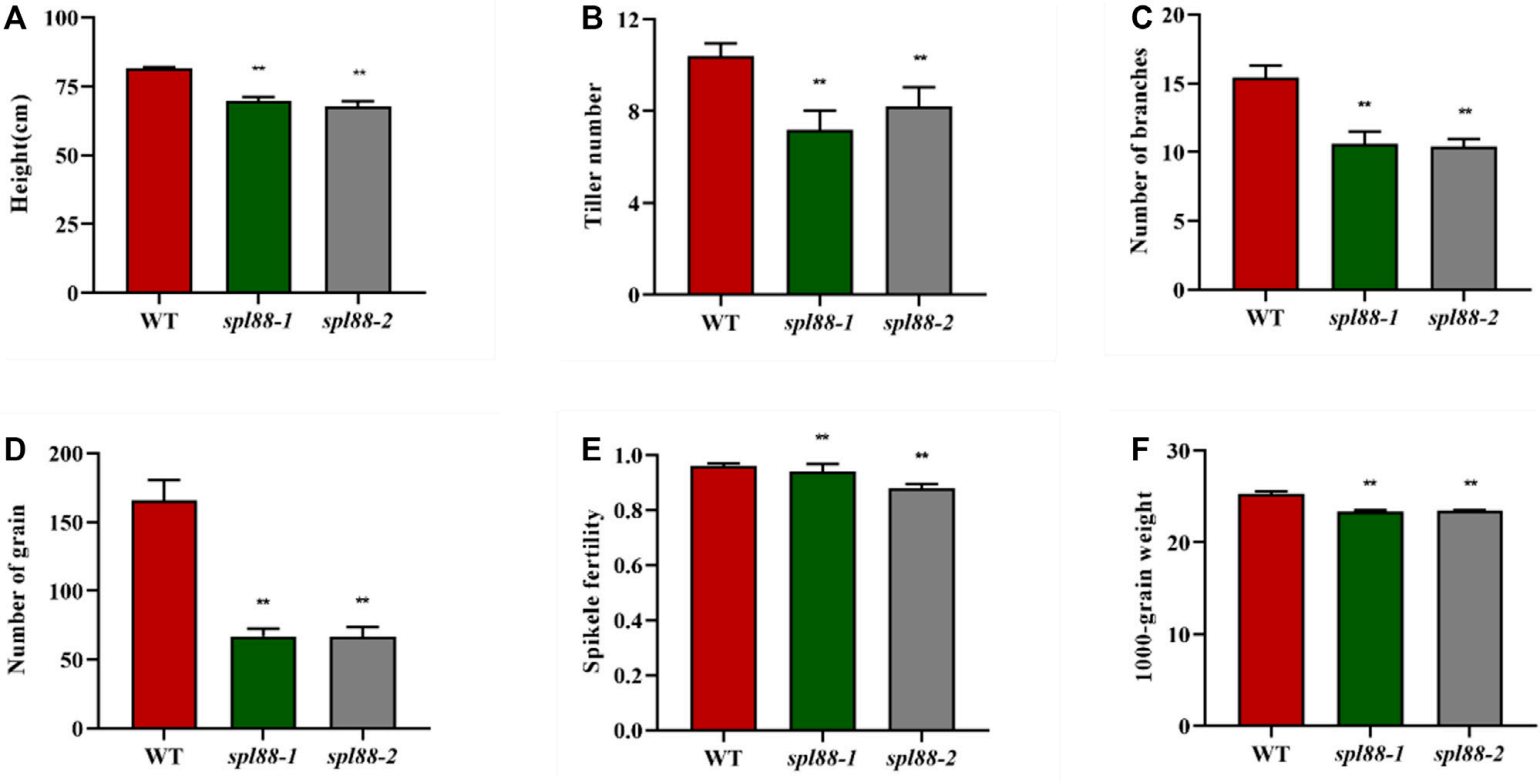
Main agronomic traits of wild-type and mutant plants. **(A)**: plant height; **(B)**: tiller number; **(C)**: number of branches; **(D)**: number of grain; **(E)**: spikele fertility; **(F)**: 1000-grain weight; values are means ± SD (n = 10); * indicates significance at *p* ≤ 0.05 by Student’s *t* test; ** indicates significance at *p* ≤ 0.01 by Student’s *t* test.

The main agronomic traits of WT, *spl88-1*, and s*pl88-2* were statistically analyzed at the booting and maturity stages. Compared with those of WT, plant height, tiller number, number of branches, number of grains, 1000-grain weight, and seed setting rate of *spl88-1* and *spl88-2* were significantly decreased (Figures 2A–F).

### *SPL88* Regulates Plant Growth and Development

**FIGURE 3:**
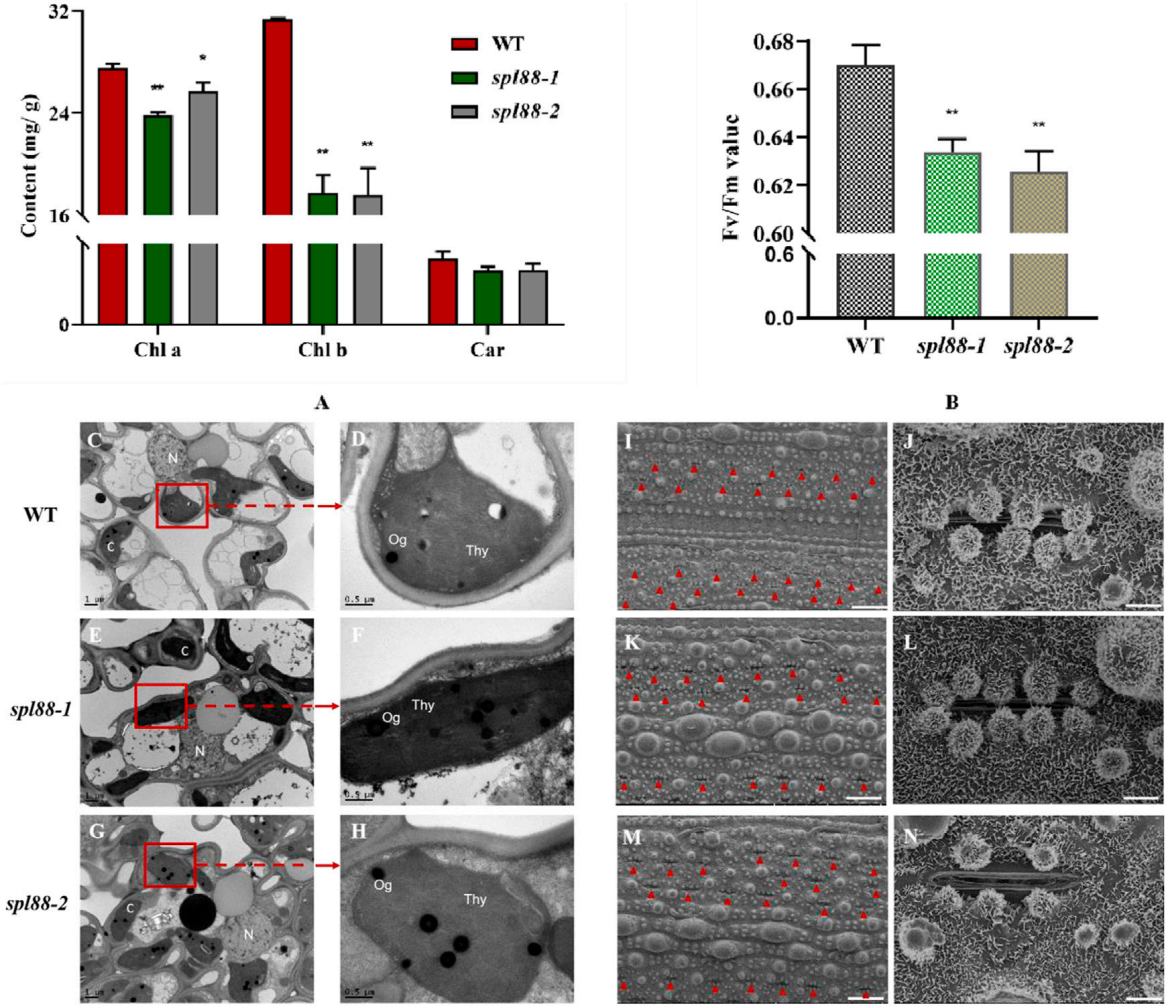
Chloroplast development, maximum photosynthetic rate, and the observation of mesophyll cell electron microscope in wild-type and mutant plants. **(A)** Chlorophyll content in flag leaves of wild-type and mutant plants. **(B)** Maximum photosynthetic rate of wild-type and mutant plants. **(C–H**) Ultrastructural observation of chloroplast of wild type and mutant plants; **(N)**: nucleus; Thy: chloroplast; Og: osmium granules; Bar = 1 μm. **(I–N)**: observation of stomatal pore diameter of wild-type and mutant plant leaves; I, **(K,M)**: Bar = 300 μm; **(J,L,N)**: Bar = 30 μm * indicates significance at *p* ≤ 0.05 by Student’s *t*-test; ** indicates significance at *p* ≤ 0.01 by Student’s *t*-test.

We speculated that the lesion mimic phenotype of *spl88-1* and *spl88-2* affected the growth and development of plants. The chlorophyll content of WT, *spl88-1*, and *spl88-2* was determined at the tillering stage. The results showed that the carotenoid content of *spl88-1* and *spl88-2* did not change significantly, while chlorophyll a and chlorophyll b were significantly lower than those in WT, and the chlorophyll a of *spl88-1* decreased more significantly (Figure 3A). Compared with WT, the maximum photosynthetic rates of *spl88-1* and *spl88-2* were significantly decreased (Figure 3B). In addition, we observed the ultrastructure of chloroplasts by transmission electron microscope and found that the chloroplasts of *spl88-1* and *spl88-2* were shrunken and the internal lamella structure was disordered. More obviously, the osmium granules in the chloroplasts of *spl88-1* and *spl88-2* were obviously increased (Figures 3C–H). Scanning electron microscopy (SEM) results showed that the stomatal pore sizes of *spl88-1* and *spl88-2* were significantly larger than those of WT (Figure 3I–N). The aforementioned results indicate that the changes in chloroplast structure of *spl88-1* and *spl88-2* affect the growth and development of plants.

### *SPL88* Regulates ROS Accumulation and Cell Death in Rice

**FIGURE 4:**
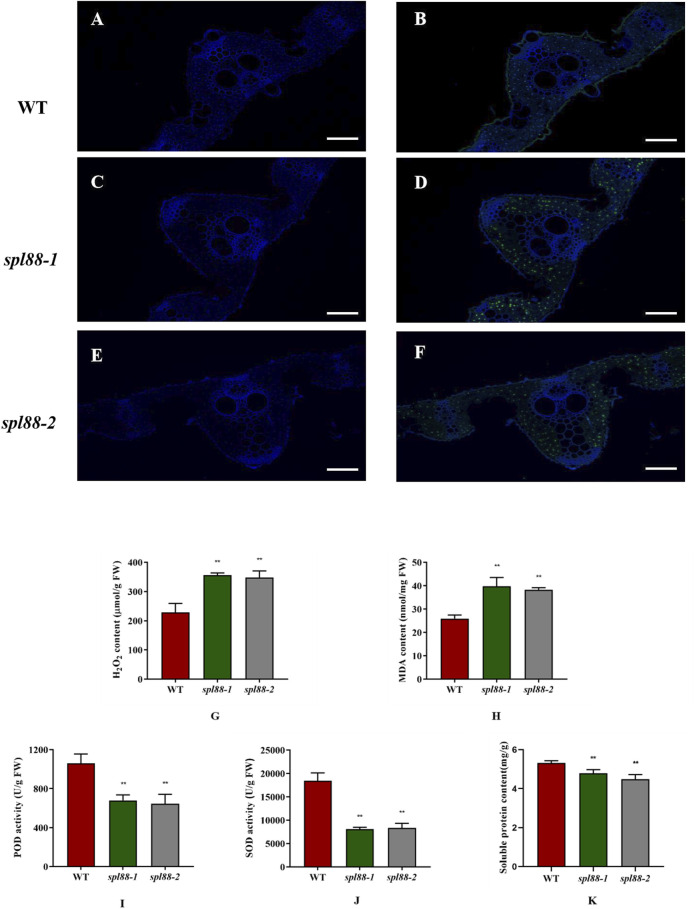
Physiological and biochemical analysis of wild-type and mutant plants at the tillering stage. **(A–F)**: TUNEL assay of DNA fragmentation in mesophyll cells; Bar: 200 μm. **(G–K)**: H_2_O_2_, MDA, and soluble protein contents of leaves, POD, and SOD activities in the leaves. *indicates significance at *p* ≤ 0.05 by Student’s *t*-test, ** indicates significance at *p* ≤ 0.01 by Student’s *t*-test.

The TUNEL assay is a marker of programmed cell death (Kim et al., 2009; Wang et al., 2017), which is used to detect DNA fragmentation. To explore whether this type of lesion mimic phenotype could cause the PCD pathway of mutants *spl88-1* and *spl88-2*, we performed TUNEL signal detection. A weaker TUNEL signal was observed in WT, while the TUNEL signals of *spl88-1* and *spl88-2* were significantly enhanced, and the fluorescence signal distribution was random (Figures 4A–F). When plants are under stress or senescence, high concentrations of ROS will accumulate, leading to formation of H_2_O_2_, which directly or indirectly oxidizes biological macromolecules such as nucleic acids, proteins, and lipids, thereby destroying the cell membrane structure and causing programmed cell death (Chen et al., 2018; Hoang et al., 2019). POD can catalyze the direct oxidation of H_2_O_2_ into phenolic or amine compounds to eliminate the toxic effect of H_2_O_2_ (Rechcigl and Evans, 1963); SOD can eliminate superoxide anion-free radicals and plays a crucial role in anti-oxidation (Sharma et al., 20122012; Yuchun et al., 2021). Therefore, we measured H_2_O_2_ content, POD activity, and SOD activity in the plants and found that a large amount of H_2_O_2_ accumulated in *spl88-1* and *spl88-2*, while POD and SOD activities were significantly reduced, and the soluble protein content also decreased significantly. When plants accumulate large amounts of H_2_O_2_, lipid peroxidation occurs, producing large amounts of MDA (Sun et al., 2017; Tu et al., 2020). The MDA content in *spl88-1* and *spl88-2* was significantly higher than that in WT (Figures 4G–K).

### *SPL88* Regulates Defense Responses in Rice

**FIGURE 5:**
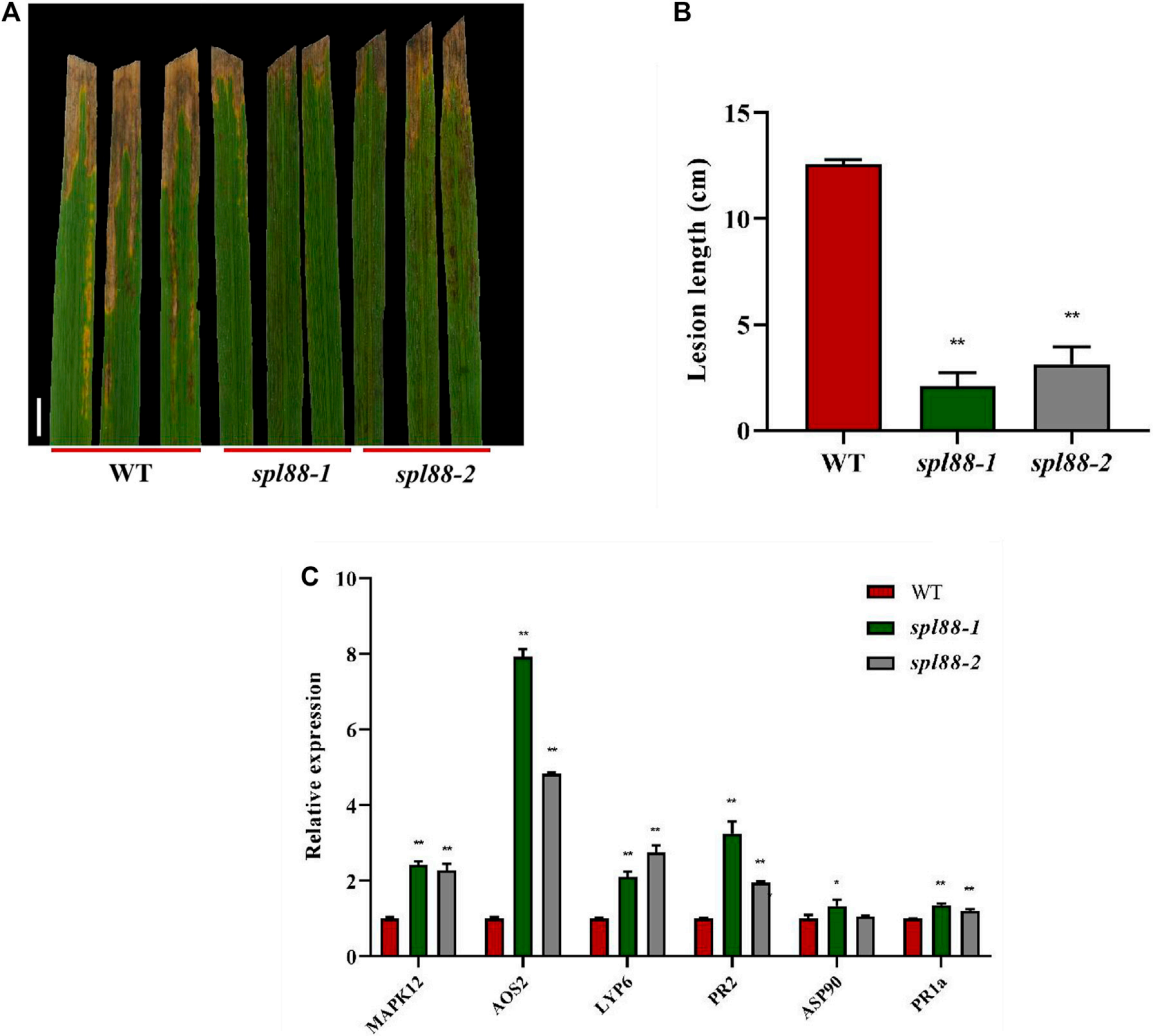
*SPL88* regulates defense responses in rice. **(A)** Phenotype of wild-type and mutant plant leaves at 15 days after inoculation with the bacterial blight pathogen HM73, Bar = 3 cm; **(B)** statistical analysis of the length of bacterial leaf blight lesions; **(C)** relative expression of defense-related genes in plants; *indicates significance at *p* ≤ 0.05 by Student’s *t* test, **indicates significance at *p* ≤ 0.01 by Student’s *t* test.

Most rice lesion mimic mutants show resistance to fungal and bacterial pathogens. To investigate whether *spl88-1* and *spl88-2* also have this resistance, we used the leaf clipping method (Zang et al., 2000) to inoculate WT, *spl88-1*, and *spl88-2* with the bacterial blight strain HM73. After 15 days of inoculation, the phenotype was observed and the length of the lesions was measured. The length of bacterial leaf blight lesions of WT was significantly longer than that of *spl88-1* and *spl88-2* (Figures 5A,B). This indicated resistance to the *Xanthomonas oryzae pv. Oryzae*, *Xoo* was significantly enhanced after the emergence of disease spots in *spl88-1* and *spl88-2*. In addition, we detected the expression of defense-related genes in WT, *spl88-1*, and *spl88-2* by qRT-PCR. The expression levels of defense-related genes *MAPK12*, *AOS2*, *LYP6*, *PR2*, *ASP90*, and *PR1a* in *spl88-1* and *spl88-2* were significantly increased (Figure 5C). Among them, the expression of *AOS2* increased most obviously, and *SPL88* may respond to the damage caused by the lesion mimics by inducing the synthesis of JA (Kemal and Manners, 2011; Wakuta et al., 2011). These results show that the loss of function of the *SPL88*-encoded protein triggers a defense response in rice, leading to enhanced resistance to pathogens in *spl88-1* and *spl88-2*.

### Genetic Analysis and Map-Based Cloning of *SPL88*

We used *spl88-1* and *spl88-2* to cross with the japonica cultivar Nanjing 06 to get F_1_. The F_1_ plants did not show lesion mimics. F_2_ generation plants appeared similar in phenotype to *spl88-1* and *spl88-2*. The segregation ratio of lesion mimic phenotype to the normal phenotype in the F_2_ population was essentially in compliance with a 1:3 ratio (Supplementary Table S4, S5), indicating that the *spl88-1* and *spl88-2* phenotype was caused by a mutation in a single recessive nuclear gene.

**FIGURE 6:**
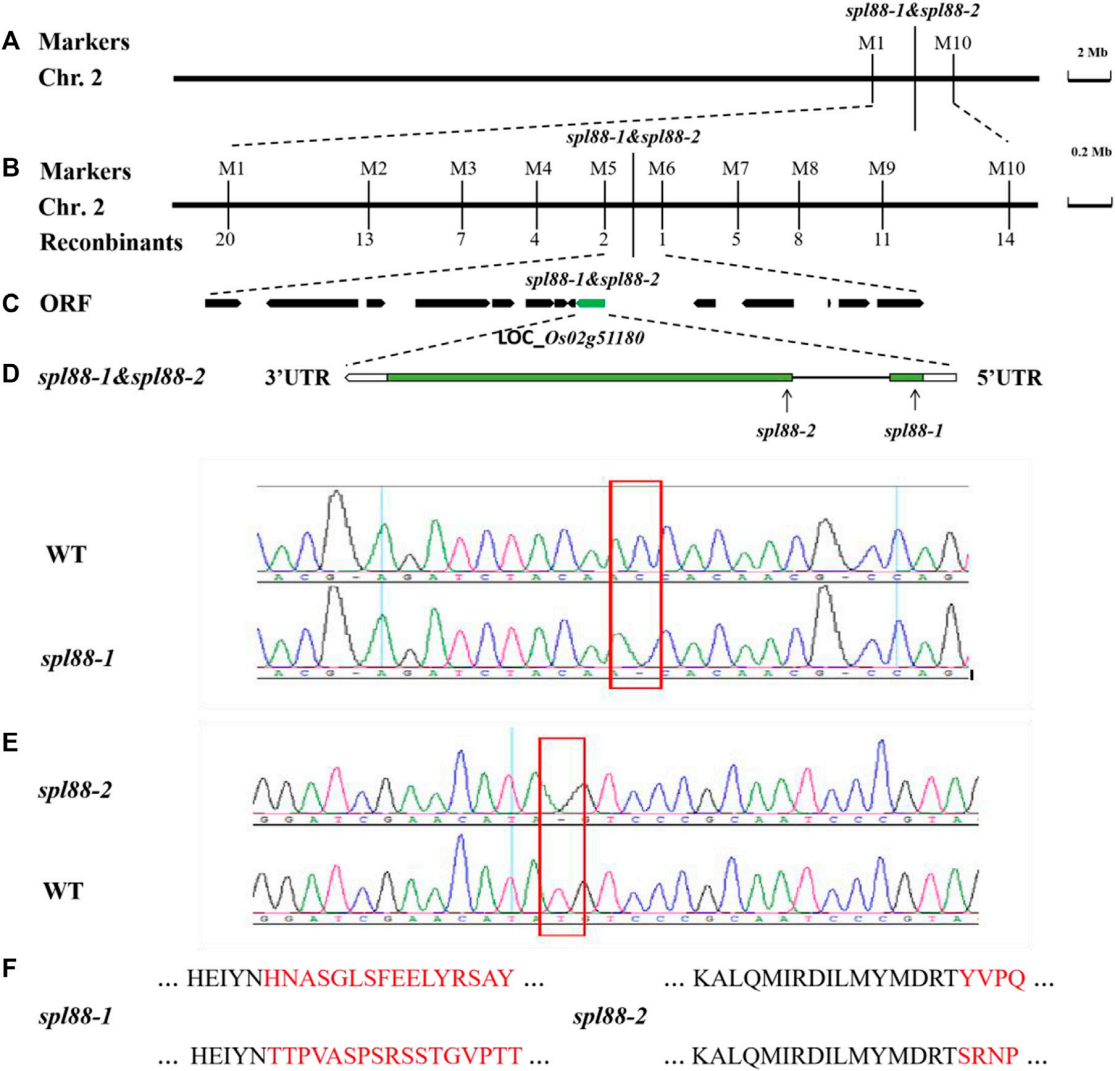
Genetic and physical maps of the *SPL88*. **(A)** *SPL88* gene was localized to chromosome 2 between InDel markers M1 and M10; **(B)** *SPL88* gene was delimited to the M5 to M6 interval on chromosome 2; **(C)** 14 putative ORFs located in the ∼84 kb region of this locus; **(D)** gene structure of *LOC_Os02g51180*; **(E)** sequence analysis of C-base deletion mutation sites in wild-type and *spl88-1*; sequence analysis of T-base deletion mutation sites in wild-type and *spl88-2*; **(F)** mutation causes premature termination of protein translation.

We used 238 pairs of SSR molecular markers to map the mutant gene in 21 F_2_ individuals with lesion mimic phenotype and preliminarily located the mutation site between the two molecular markers M1 and M10 on the long arm of chromosome 2. Further through the indica–japonica difference, we developed four newly polymorphic tags and ultimately mapped *SPL88* to an 84-kb region between markers M5 and M6. It was predicted that this region contains 14 open-reading frames (ORFs) by http://rice.uga.edu/. Sequencing found that the gene *LOC_Os02g51180* was mutated, in which the 132nd base C in the coding region of the *SPL88-1* gene was deleted, and the 381st base T in the coding region of the *SPL88-2* gene was deleted, resulting in early termination (Figures 6A–F). Therefore, *LOC_Os02g51180* is the candidate gene for *SPL88*.

To verify that the phenotype of mutants *spl88-1* and *spl88-2* was caused by the deletion of the coding region of the gene *LOC_Os02g51180*, we constructed complementary vectors *pGSPL88-1* and *pGSPL88-2*, respectively. The two vectors contained genomic DNA fragments including the promoter of the *SPL88* gene in WT Xiushui 11 and were introduced into *spl88-1* and *spl88-2* by *Agrobacterium tumefaciens*–mediated transformation (Zhang and Chen, 2007; WangShen et al., 2011). Among the transformed T_0_ generation plants, the positive plants were consistent with the phenotype of WT and the negative plants were consistent with the lesion mimic phenotype of mutants *spl88-1* and *spl88-2* (Supplementary Figure S1). The aforementioned results verify the accuracy of the candidate gene *LOC_Os02g51180* and also prove that the lesion phenotype of *spl88-1* and *spl88-2* is caused by the single-base deletion of the gene *LOC_Os02g51180*.

### Expression Analysis of *SPL88*

**FIGURE 7:**
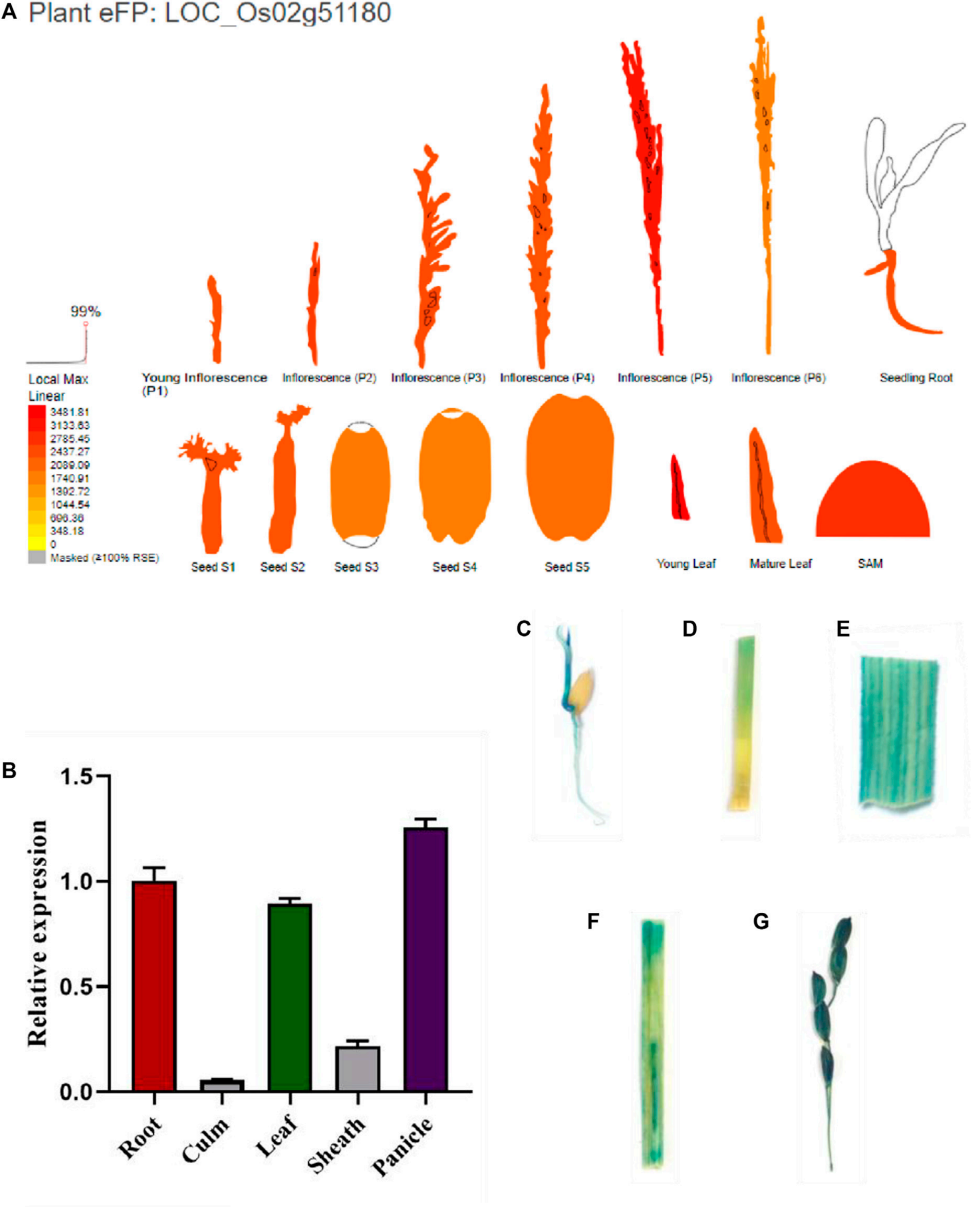
Expression analysis of *SPL88*. **(A)** Site predicts the expression pattern of *SPL88*; **(B)** expression of *SPL88* in various organs of wild-type and mutant plants analyzed by qRT-PCR; **(C–G)** histochemical signals from the *SPL88* promoter-GUS reporter gene. GUS signals were detected in root C, stem D, leaf E, leaf sheath F, and panicle **(G)**

Using http://bar.utoronto.ca/eplant_rice/to predict the expression patterns of *SPL88* in various organs, we found that *SPL88* was mainly expressed in root, leaves, and panicle, especially high in leaves and panicle (Figure 7A). To verify this prediction, we constructed the vector *pSPL88::GUS*, which contained promoter fragments of the *SPL88* and introduced it into WT by *Agrobacterium tumefaciens*–mediated transformation. We stained various organs of the transgene-positive plants and observed GUS signals in various tissues. In addition, we used qRT-PCR to analyze the expression of *SPL88* in various organs and found that the results were consistent with the results of GUS staining, with the highest expression in panicle and the lowest expression in the stem.

### Subcellular Localization of *SPL88*

**FIGURE 8:**
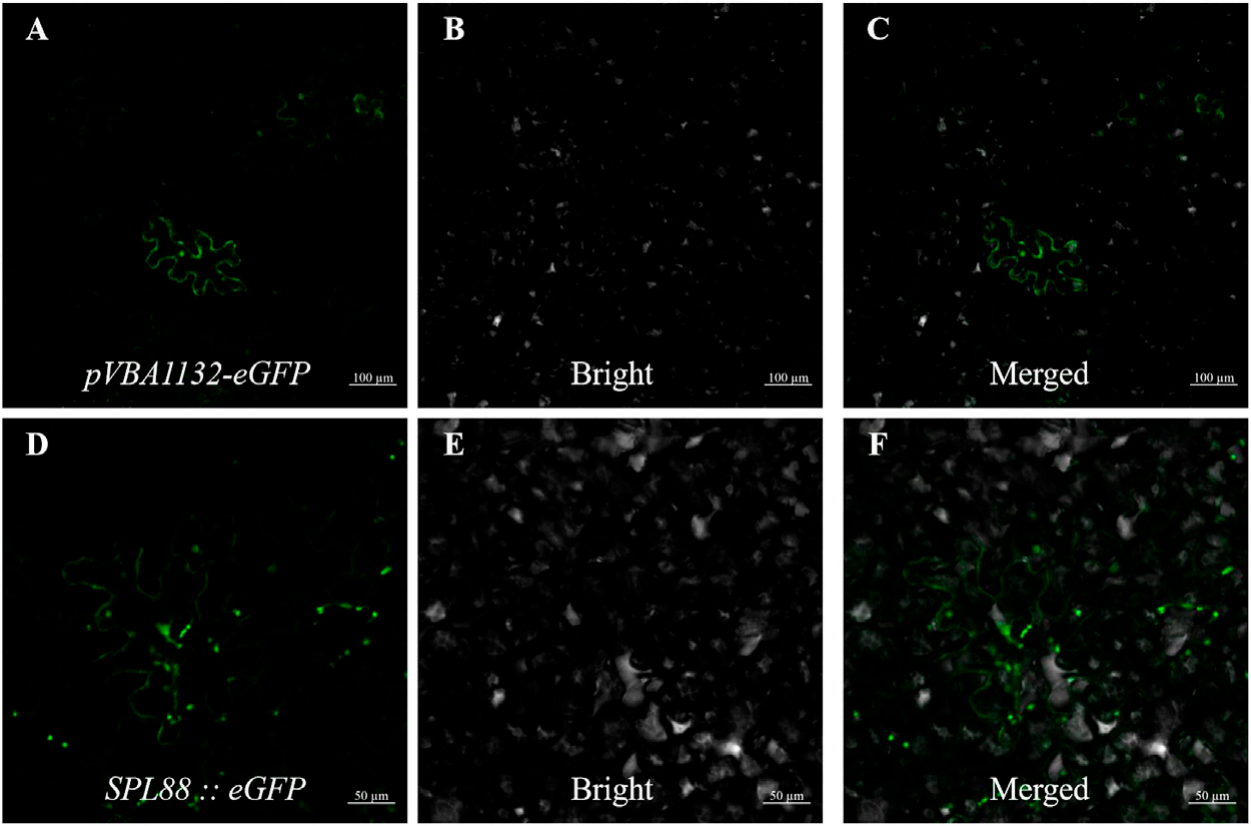
Subcellular localization of *SPL88*; **(A–C)**: transient expression of *pVBA1132-eGFP* in tobacco, Bar: 100 μm; **(D–F)**: transient expression of *pVBA1132:SPL88::eGFP* in tobacco, Bar: 50 μm.

To explore the localization pattern of *SPL88* in cells, we constructed the *pVBA1132::SPL88::eGFP* vector, which contained the *SPL88* coding sequence of WT and was transiently expressed in tobacco epidermal cells as observed by laser scanning confocal microscopy (CLSM). There are strong green fluorescence signals in the cytoplasm and nucleus, indicating that *SPL88* is expressed in the cytoplasm and nucleus (Figures 8A–F).

### Transcriptome Analysis Suggested That *SPL88* Regulates Defense Responses in Rice

**FIGURE 9:**
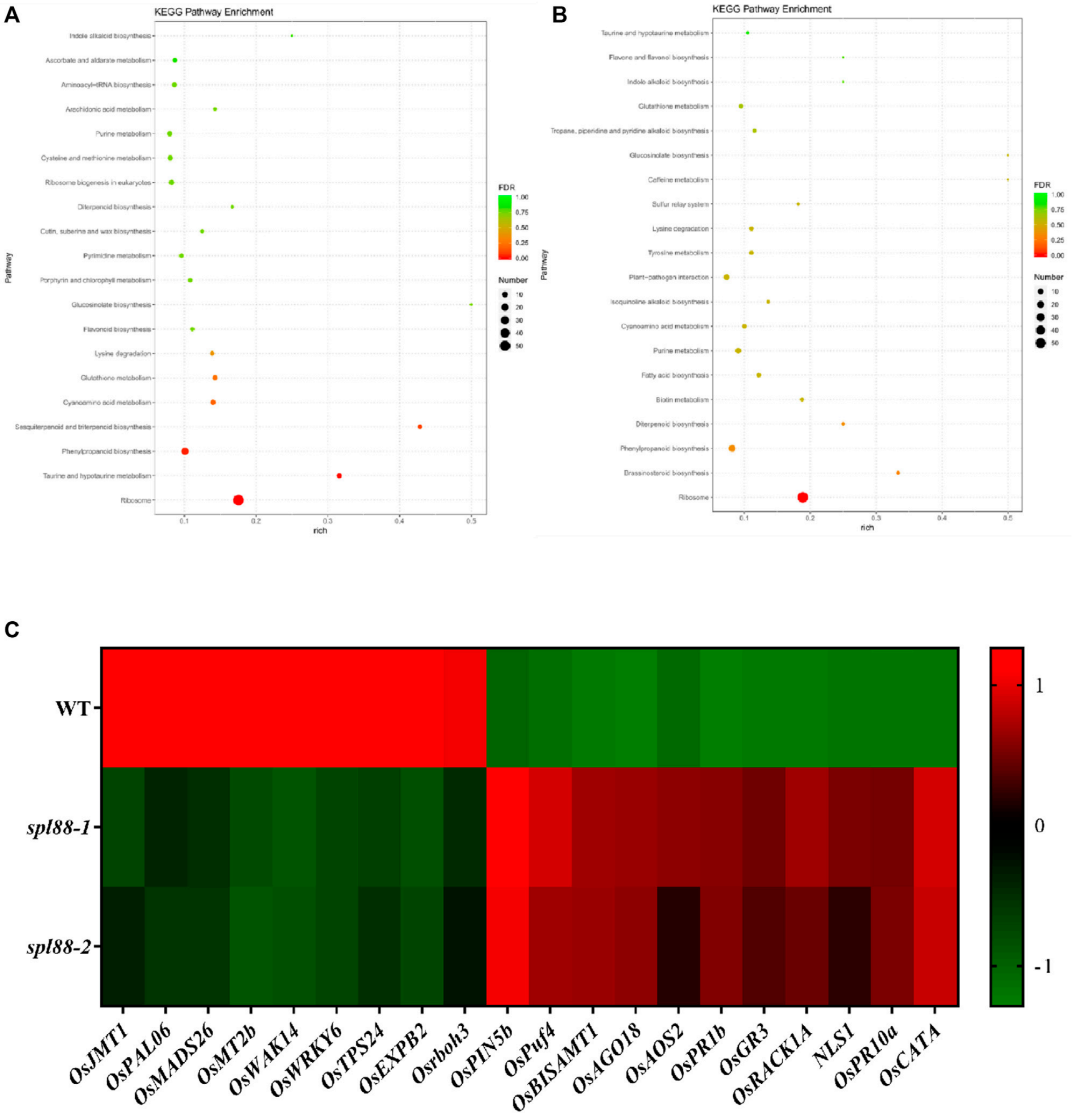
Transcriptome profiling analysis of *spl88-1* and *spl88-2*. **(A)**, **(B)** KEGG enrichment analysis of differentially expressed genes compared to wild type, *spl88-1*, and *spl88-2*; **(C)** expression heat map of differentially expressed genes associated with defense and growth and development.

To investigate the potential regulatory mechanisms of lesion mimics in the *spl88-1* and *spl88-2*, we sampled flag leaves from WT, *spl88-1*, and *spl88-2* at the tillering stage with the most necrotic spots and performed transcriptome sequencing to compare global gene expression changes between *spl88-1*, *spl88-2*, and WT. The result revealed that a total of 1564 differentially expressed genes were found in *spl88-1*, of which 788 genes were upregulated (log2 ratio ≥ 1) and 766 were downregulated (log2 ratio ≤ −1). 531 genes were upregulated and 998 were downregulated in *spl88-2*. According to the Kyoto Encyclopedia of Genes and Genomes (KEGG) enrichment analysis, the differentially expressed genes were mainly involved in signaling pathways and physiological metabolic pathways, including indole alkaloid biosynthesis, purine metabolism, diterpenoid biosynthesis, phenylpropanoid biosynthesis, taurine, and hypotaurine metabolism and ribosome synthesis (Figures 9A,B). Based on the RNA-seq results, the defense-related genes and growth and development-related genes including *OsJMT1*, *OsPAL06*, *OsMADS26*, *OsMT2b*, *OsWAK14*, *OsWRKY6*, *OsTPS24*, *OsEXPB2*, *Osrboh3*, *OsPIN5b*, *OsPuf4*, *OsBISAMT1*, *OsAG O 18*, *OsAOS2*, *OsPR1b*, *OsGR3*, *OsRACK1A*, *NLS1*, *OsPR10a*, *OsCATA* were significantly differentially expressed between *spl88-1*, *spl88-2*, and WT (Figure 9C). These results suggest that *SPL88* plays an important role in the defense response in rice.

## Discussion

Lesion mimics often affect normal growth and development in plants, resulting in weakened photosynthesis and reduced yield. Therefore, the research on rice lesion mimic mutants is of great significance for elucidating the mechanism of plant growth and development. In this study, we obtained two lesion mimic mutants *spl88-1* and *spl88-2*. At the tillering stage, the lesion mimic phenotype appeared in the leaf apex of *spl88-1* and *spl88-2*. With the growth of the plants, the necrotic spots gradually spread throughout the leaves. A transmission electron microscope showed that the chloroplast ultrastructure of mutants *spl88-1* and *spl88-2* changed, which affected the growth and development of plants. Furthermore, through a series of physiological and biochemical analyses, we found that the appearance of lesion mimic phenotype led to the accumulation and burst of ROS in mutants *spl88-1* and *spl88-2*, resulting in cell death. The gene *LOC_Os02g51180* was confirmed to be the candidate gene of *SPL88* by map-based cloning and genetic complementation experiments, in which the 132nd base C in the coding region of the *SPL88-1* gene was deleted, and the 381st base in the coding region of the *SPL88-2* gene had a T deletion, resulting in premature termination of SPL88 translation and formation of three distinct proteins (Supplementary Figure S2). We used the website (http://smart.embl-heidelberg.de/) to analyze the protein sequence encoded by *SPL88* and found that the protein contained two domains of CULLIN and Cullin Nedd8 (Supplementary Figure S3). The CULLIN domain consists of 147 (407-554) amino acids and the Cullin Nedd8 domain consists of 67 (663-730) amino acids. According to the website (https://services.healthtech.dtu.dk/service.php?TMHMM-2.0), it was predicted that the 736 amino acids of the protein were located on the surface of the cell membrane and did not form a typical transmembrane helical region, which did not belong to transmembrane protein (Supplementary Figure S4). To explore whether *SPL88* is involved in the pathways related to adversity stress, we conducted a hydroponic salt stress experiment on the wild type, *spl88-1*, and *spl88-2.* We treated the seedlings in the trefoil stage with 150 mM/L NaCl for 4 days and found that the phenotype of wild-type and mutant plants did not change obviously before and after the treatment, indicating that *SPL88* did not involve in the response to salt stress (Yan et al., 2021) (Supplementary Figure S5).

Although the lesion mimic phenotypes of *spl88-1* and *spl88-2* were both caused by deletion of the same gene and the series of positive and negative effects brought about by the lesion mimic phenotype were generally consistent, there were still some subtle differences. In the chlorophyll assay results, the chlorophyll a of *spl88-1* decreased more and the chloroplasts were more severely shrunken, while the chloroplasts of *spl88-2* were more inclined to chloroplast disorder and the expression levels of defense-related genes *AOS2* and *ASP90* in *spl88-1* were also significantly higher than those in *spl88-2*. According to these results, we speculate that the aforementioned results are because the *SPL88-1* base is deleted in the first exon and the *SPL88-2* base is deleted in the second exon, but not within the CULLIN and Cullin Nedd8 domains, and both of the two domains do not express.

We confirmed that the candidate gene *SPL88* indeed controlled the phenotype of mutants *spl88-1* and *spl88-2* by map-based cloning and complementation experiments, indicating that *SPL88* was a new allelic variant gene of cloned *OsCUL3a*, which interacts with *OsRBX1a* and *OsRBX1b* to form RING E3 ubiquitin ligase (CRL3) involved in plant immune responses (Liu et al., 2017; Gao et al., 2020). Like *oscul3a*, *spl88-1* and *spl88-2* exhibited enhanced defense responses and reduced seed setting. The difference was that we found *SPL88* not only damaged the chloroplast structure but also hindered chlorophyll biosynthesis. We also detected many apoptotic cells in *spl88-1* and *spl88-2* by TUNEL assay. In addition, we demonstrated *SPL88* was expressed in all organs and expressed the most in roots and leaves. Based on the aforementioned analysis, we speculate *SPL88* is a different mutant form from the reported allele of *OsCUL3a*.

Mutants are important materials for studying gene function. For the study of the gene regulatory network of lesion mimics, it is necessary to strengthen the screening and gene function identification of different types of lesion mimic mutants in the future. The research objects *spl88-1* and *spl88-2* are of great significance for mining and analyzing the regulation mechanism of lesion mimics and improving the regulation network of lesion mimics.

## Data Availability

The datasets presented in this study can be found in online repositories. The names of the repository/repositories and accession number(s) can be found in the article/Supplementary Material.
