# Supplementary material for: OsSPL88 Encodes a Cullin Protein that Regulates Rice Growth and Development
Source: Front Genet. 2022 Jul 11;13:918973. doi: 10.3389/fgene.2022.918973 (PMC9309799; doi:10.3389/fgene.2022.918973)
Supplement: Supplementary file 1 [file DataSheet2.PDF]

Supplementary Material

Supplementary Table 1 Primers used for qRT-PCR

Primer name	Forward primer (5′→3′)	Reverse primer (5′→3′)
OsActin	TGGCATCTCTCAGCACATTCC	TGCACAATGGATGGGTCAGA
MPK12-qRT	TGACCAAGAGAGGAGTGCAG	CGTCATCGTTGTGCACTAGG
AOS2-qRT	GAGAGACGGAGAACCCTAGC	GAAGTGATGGCCGGCTTAAG
LYP6-qRT	AACTGCTGGAAATGTGTGC	TTGAAGACCAGAGGAGAGACG
PR2-qRT	CGGTACAAGTAGGAGGAGCT	GCTCGACGTTGAACCTGATC
ASP90-qRT	CCTTCCAACTGAGGTCGAGT	AGACTGCAGGCTGTGTAAGA
PR1a-qRT	TCTCACCAGCATACGTCGT	ATCCCAAGTCCTGCGTACAA

Supplementary Table 2 Primers used for mapping

Primer name	Forward primer (5′→3′)	Reverse primer (5′→3′)
M1	CGCTGCTCACCGTCACTG	GCAAAGCAATCGCAGAATTT
M2	CCTCTTCCTCCCACTCTCCT	GTCGTCGTCGTACTCCTCGT
M3	GCCATGTCATATTCCTATGCAA	GGGTGAATTAGACATAACCATAGG
M4	ACTCCCTCCGTTTCATAATGT	TGTGTATGATAGGTGGGACCAG
M5	TCGTGGTTAGTAAGCTTCCATGT	CGAACCAATGGCTTCTTGAC
M6	TTTTTCGTCACGTCAAATGTT	CCTGTAAACAGCGAGACGAA
M7	TGGAGAGTAAATGGGGGAAT	CCGCTGGTTCATCAAGTCTA
M8	CTGATGGGCGAACAACCT	CTTGGTCGTCGTCACTGTTG
M9	GCATGCCACAAATCGTTGTA	TCGCCATTCGTGTGACTACT
M10	GGCATAAATTAGGCGCAAAG	TTTTACCGTGTTGGTGCAAA

Supplementary Table 3 Primers used for vector construction

Primer name	Primer sequence (5′→3′)
SPL88-1300-F	CATGATTACGAATTCGCTGCACATGGTCAAACTGA
SPL88-1300-R	GCCAGTGCCAAGCTTGGCTGTGAATTTGACGATCCAT
SPL881305.1-F	CATGATTACGAATTCGCTGCACATGGTCAAACTGA
SPL88-1305.1-R	TCAGATCTACCATGGCGCCGCCGCCGGTGAGGTC
SPL88-1132-F	TTCGATATCAAGCTTATGAGCGGGGGCGGG
SPL88-1132-R	GCTCACCATGGTACCTGCAAGATAGCGATATAACTTCC

Supplementary Table 4 *spl88-1* genetic analysis

Cross	F_1 phenotvpe	F_2 phenotvpe			χ^2（3：1）
		Normal	Lesion mimics	Total	
spl88-1/NJ06	normal	681	231	912	0.0526
NJ06/*spl88-1*	normal	594	193	787	0.0953

Supplementary Table 5 *spl88-2* genetic analysis

Cross	F_1 phenotvpe	F_2 phenotvpe			χ^2（3：1）
		Normal	Lesion mimics	Total	
spl88-2/NJ06	normal	573	154	627	0.0643
NJ06/*spl88-2*	normal	659	215	874	0.0748

Supplementary Figure 1 Functional Complementation of the *spl88-1* and *spl88-2* Mutants with *LOC_Os02g51180*

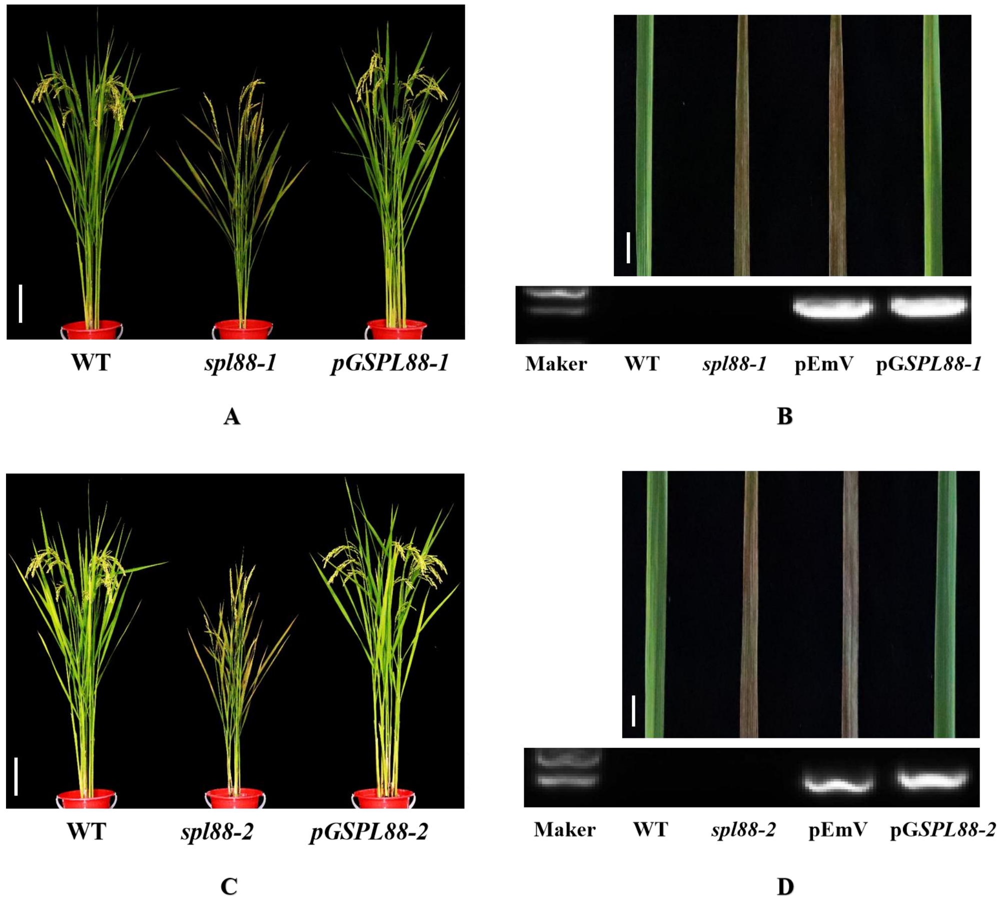

A, C: the phenotype of the *spl88-1* and *spl88-2* transformed with the genomic sequence of *SPL88* (pGSPL88) was completely recovered to that of the wild type; Bar=15 cm.

B, D: hygromycin selection of transgenic plants. pEmV: empty vector; Bar=3 cm.

Supplementary Figure 2 Three-dimensional structure of SPL88, SPL88-1 and SPL88-2

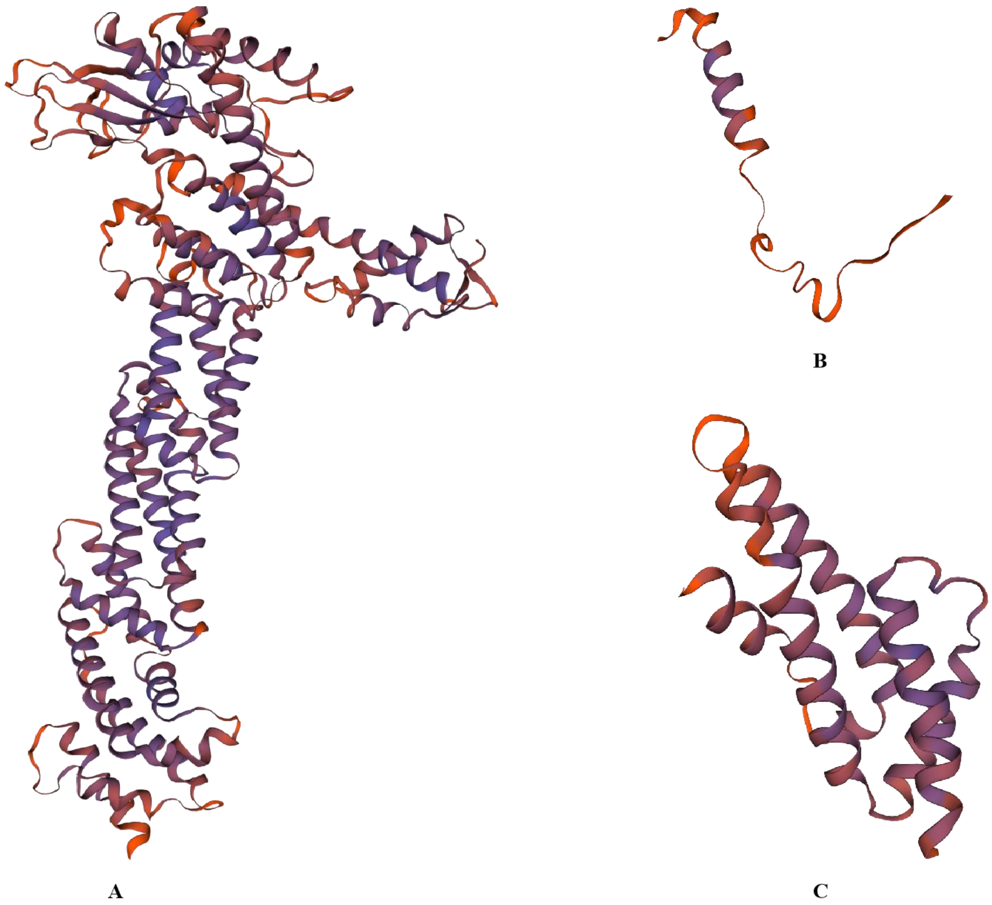

A: SPL88; B: SPL88-1; C: SPL88-2

Supplementary Figure 3 Structural prediction of the protein encoded by *SPL88*

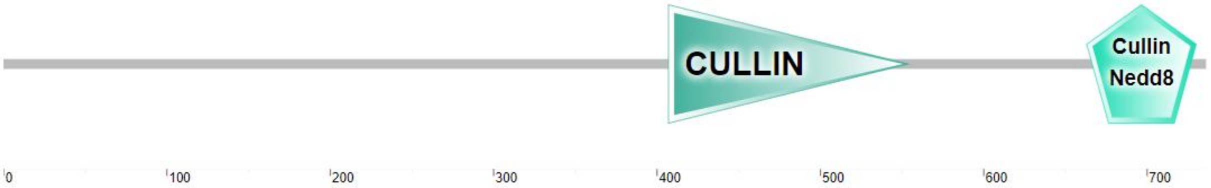

Supplementary Figure 4 *SPL88* transmembrane domain analysis

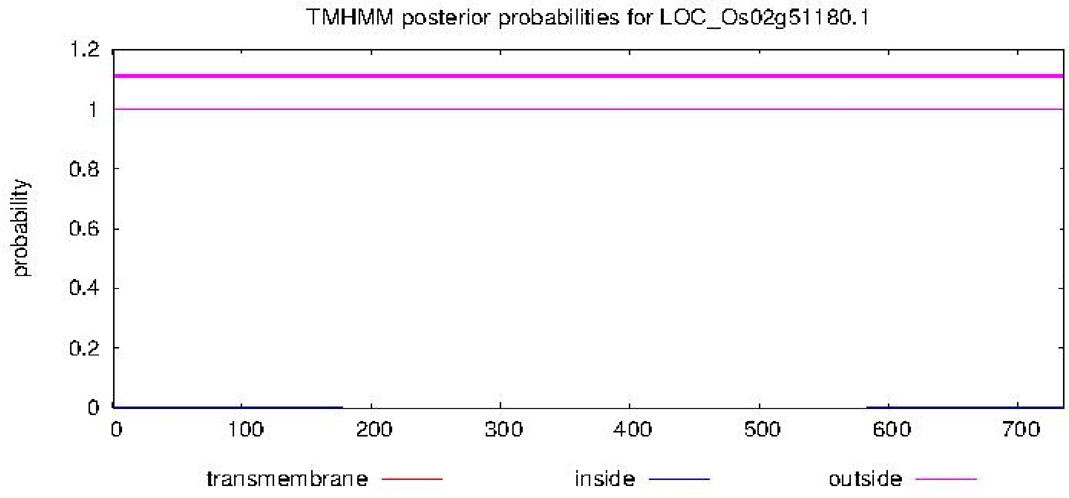

Supplementary Figure 5 Analysis of salt stress in wild type, *spl88-1* and *spl88-2*

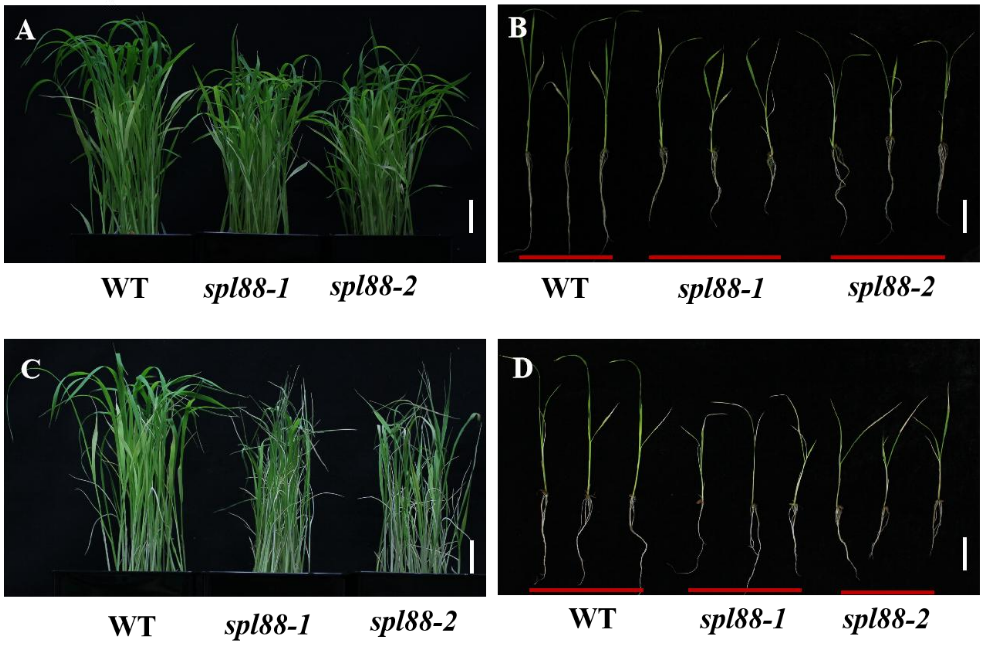

A, B: Wild-type and mutant plants on 0 mM NaCl at 4 days of culture.
C, D: Wild-type and mutant plants on 150 mM NaCl at 4 days of culture.
