# Supplementary material for: OsSPL88 Encodes a Cullin Protein that Regulates Rice Growth and Development
Source: Front Genet. 2022 Jul 11;13:918973. doi: 10.3389/fgene.2022.918973 (PMC9309799; doi:10.3389/fgene.2022.918973)
Supplement: Supplementary file 2 [file DataSheet1.PDF]

# *Supplementary Material*

## Supplementary Table 1 Primers used for qRT-PCR

| Primer name | Forward primer (5′→3′) | Reverse primer (5′→3′) |
|---|---|---|
| *OsActin* | TGGCATCTCTCAGCACATTCC | TGCACAATGGATGGGTCAGA |
| *MPK12*-qRT | TGACCAAGAGAGGAGTGCAG | CGTCATCGTTGTGCACTAGG |
| *AOS2*-qRT | GAGAGACGGAGAACCCTAGC | GAAGTGATGGCCGGCTTAAG |
| *LYP6*-qRT | AACTGCTGGAAATGTGTGC | TTGAAGACCAGAGGAGAGACG |
| *PR2*-qRT | CGGTACAAGTAGGAGGAGCT | GCTCGACGTTGAACCTGATC |
| *ASP90*-qRT | CCTTCCAACTGAGGTCGAGT | AGACTGCAGGCTGTGTAAGA |
| *PR1a*-qRT | TCTCACCAGCATACGTCGT | ATCCCAAGTCCTGCGTACAA |

## Supplementary Table 2 Primers used for mapping

| Primer name | Forward primer (5′→3′) | Reverse primer (5′→3′) |
|---|---|---|
| M1 | CGCTGCTCACCGTCACTG | GCAAAGCAATCGCAGAATTT |
| M2 | CCTCTTCCTCCCACTCTCCT | GTCGTCGTCGTACTCCTCGT |
| M3 | GCCATGTCATATTCCTATGCAA | GGGTGAATTAGACATAACCATAGG |
| M4 | ACTCCCTCCGTTTCATAATGT | TGTGTATGATAGGTGGGACCAG |
| M5 | TCGTGGTTAGTAAGCTTCCATGT | CGAACCAATGGCTTCTTGAC |
| M6 | TTTTTCGTCACGTCAAATGTT | CCTGTAAACAGCGAGACGAA |
| M7 | TGGAGAGTAAATGGGGGAAT | CCGCTGGTTCATCAAGTCTA |
| M8 | CTGATGGGCGAACAACCT | CTTGGTCGTCGTCACTGTTG |
| M9 | GCATGCCACAAATCGTTGTA | TCGCCATTCGTGTGACTACT |
| M10 | GGCATAAATTAGGCGCAAAG | TTTTACCGTGTTGGTGCAAA |

**Supplementary Table 3  Primers used for vector construction**

| Primer name | Primer sequence (5′→3′) |
|---|---|
| *SPL88-1300-F* | CATGATTACGAATTCGCTGCACATGGTCAAACTGA |
| *SPL88-1300-R* | GCCAGTGCCAAGCTTGGCTGTGAATTTGACGATCCAT |
| *SPL881305.1-F* | CATGATTACGAATTCGCTGCACATGGTCAAACTGA |
| *SPL88-1305.1-R* | TCAGATCTACCATGGCGCCGCCGCCGGTGAGGTC |
| *SPL88-1132-F* | TTCGATATCAAGCTTATGAGCGGGGGCGGG |
| *SPL88-1132-R* | GCTCACCATGGTACCTGCAAGATAGCGATATAACTTCC |

**Supplementary Table 4  *spl88-1* genetic analysis**

| Cross | $F_1$ phenotvpe | $F_2$ phenotvpe | | | $\chi^2$（3：1） |
|---|---|---|---|---|---|
| | | Normal | Lesion mimics | Total | |
| *spl88-1*/NJ06 | normal | 681 | 231 | 912 | 0.0526 |
| NJ06/s*pl88-1* | normal | 594 | 193 | 787 | 0.0953 |

**Supplementary Table 5  *spl88-2* genetic analysis**

| Cross | $F_1$ phenotvpe | $F_2$ phenotvpe | | | $\chi^2$（3：1） |
|---|---|---|---|---|---|
| | | Normal | Lesion mimics | Total | |
| *spl88-2*/NJ06 | normal | 573 | 154 | 627 | 0.0643 |
| NJ06/s*pl88-2* | normal | 659 | 215 | 874 | 0.0748 |

**Supplementary Figure 1  Structural prediction of the protein encoded by *SPL88***

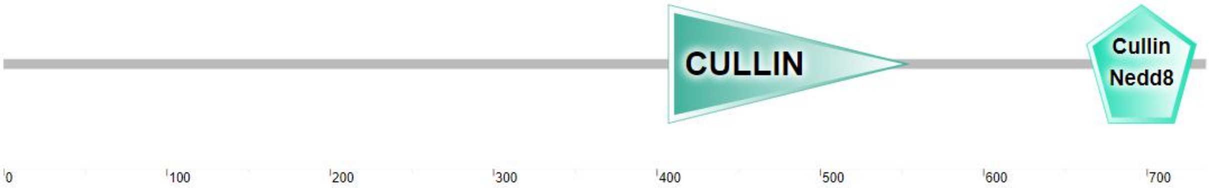


**Supplementary Figure 2  *SPL88* transmembrane domain analysis**

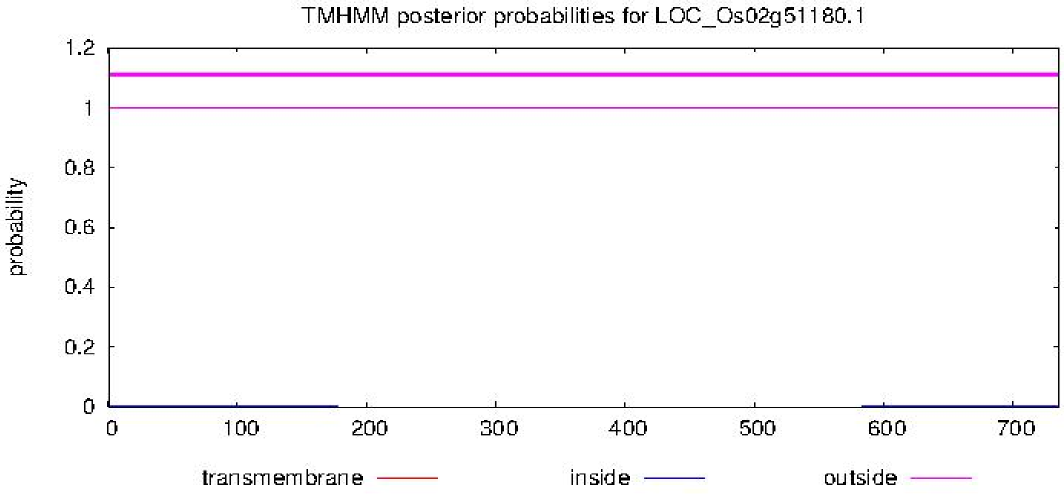


**Supplementary Figure 3  Analysis of salt stress in wild type, *spl88-1* and *spl88-2***

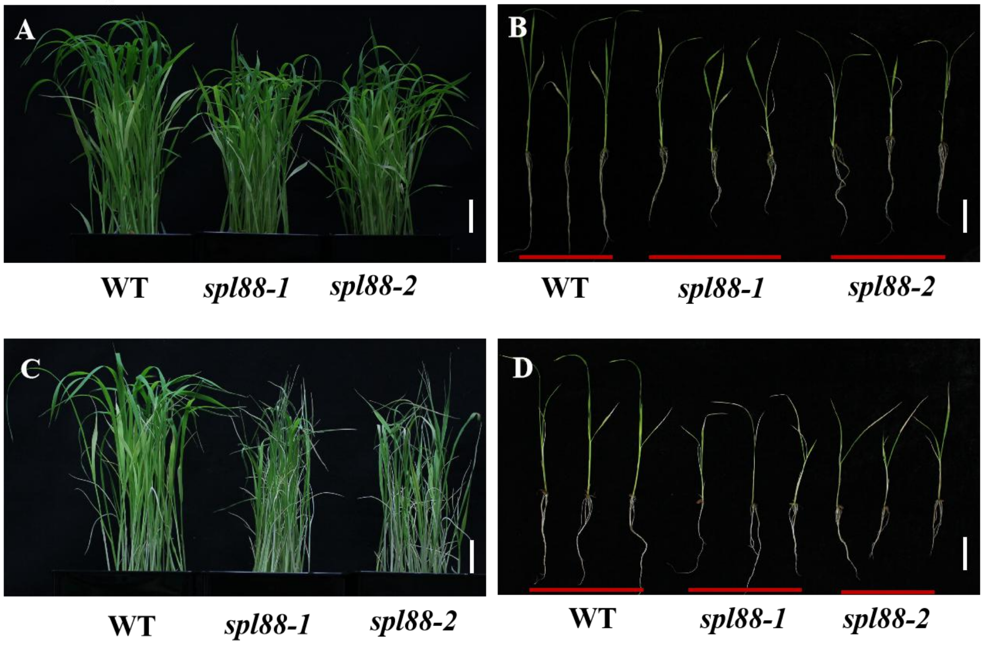


A, B: Wild-type and mutant plants on 0 mM NaCl at 4 days of culture.
C, D: Wild-type and mutant plants on 150 mM NaCl at 4 days of culture.
